# Interfacial Solar Steam/Vapor Generation for Heating and Cooling

**DOI:** 10.1002/advs.202104181

**Published:** 2022-01-12

**Authors:** Xiuqiang Li, Wanrong Xie, Jia Zhu

**Affiliations:** ^1^ Center for Advancing Electronics Dresden (cfaed) & Faculty of Chemistry and Food Chemistry Technische Universität Dresden Mommsenstrasse 4 Dresden 01062 Germany; ^2^ Department of Mechanical Engineering and Materials Science Duke University Durham NC 27708 USA; ^3^ National Laboratory of Solid State Microstructures College of Engineering and Applied Sciences Jiangsu Key Laboratory of Artificial Functional Materials and Collaborative Innovation Center of Advanced Microstructures Nanjing University Nanjing 210093 P. R. China

**Keywords:** evaporation cooling, interfacial solar steam/vapor generation, solar heating, water purification

## Abstract

Interfacial solar steam/vapor technology uses abundant and clean solar energy and water to achieve heating and cooling, a promising technology to alleviate environmental and energy issues. To obtain higher conversion and utilization efficiency, designing and optimizing materials, structures, and devices of interfacial solar steam/vapor technologies attract the attention of the research community. Given the significant progress made in the past 5 years, it is valuable to systematically summarize and discuss recent developments and future trends in this new multidisciplinary direction. This review aims to introduce interfacial solar steam/vapor principles to realize heating and cooling and the recent progress in materials, structures, devices, and applications. Meanwhile, some unsolved scientific and technical problems with outlook will also be discussed, hoping to promote further the rapid development and application of interfacial solar steam/vapor technology in heating and cooling to alleviate energy and environmental problems.

## Graphical abstract

This review systematically summarizes the development of interfacial solar steam/vapor technology in the heating and cooling fields in the past 5 years. Meanwhile, some unsolved scientific and technical problems with outlook are also discussed, hoping to promote further the development in this direction to solve the energy and environmental crises the world is facing.

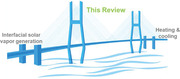

## 1 Introduction

The global heating and cooling industry size exceeds USD 1000 billion in 2020. According to forecasts, this value will have a compound annual growth rate of over 4% up to 2027 because of the implementation of several regulatory policies. For example, the growth drivers in Asia are rapid urbanization and industrialization and regulations toward sustainable energy.^[^ ^1^ ^]^ Although some clean energy technologies have been extensively developed, fossil fuels still provide most energy. According to 2019 figures from Eurostat, ≈75% of heating and cooling are derived from fossil fuels, which causes severe problems to both environment and the economy.^[^ ^2^, ^3^ ^]^ To resolve these problems, achieving high energy efficiency in heating and cooling technologies with a minimum carbon footprint has become a vital goal for sustainability. It calls for collaboration and innovation in the research community.^[^ ^4^, ^5^ ^]^

Solar steam/vapor generation as a photothermal technology plays an indispensable role in water purification, power generation, sterilization, etc. However, the traditional volumetric heating only has ≈40% solar to steam/vapor conversion efficiency.^[^ ^6^, ^7^ ^]^ Recently, the concept of interfacial solar steam/vapor generation has been proposed and achieved. Benefit from improving heat localization at the liquid surface through material design, >90% solar to vapor conversion efficiency under 1 Sun can be realized.^[^ ^8^, ^9^, ^10^, ^11^, ^12^, ^13^, ^14^, ^15^, ^16^, ^17^, ^18^, ^19^, ^20^, ^21^, ^22^, ^23^, ^24^, ^25^, ^26^, ^27^, ^28^, ^29^, ^30^, ^31^, ^32^, ^33^, ^34^, ^35^, ^36^, ^37^, ^38^, ^39^, ^40^, ^41^, ^42^, ^43^, ^44^, ^45^, ^46^, ^47^, ^48^, ^49^, ^50^, ^51^, ^52^, ^53^, ^54^, ^55^, ^56^, ^57^, ^58^, ^59^, ^60^, ^61^, ^62^, ^63^ ^]^ In the process of solar steam/vapor conversion, in addition to achieving water purification through evaporation and condensation, it is also accompanied by energy conversion. Specifically, evaporation is a process of taking away energy (e.g., the evaporation of 1 g of water needs to take away about 2260 kJ of energy at 100 °C), that is, cooling. Condensation is a process of releasing heat, that is, heating. It is not hard to see that this technology uses abundant and clean solar energy and water (which can be various forms of water) on the earth to achieve heating and cooling, a promising strategy to alleviate the energy and environmental crisis. Based on the current research results, we mainly discuss the application of heating and cooling in some commercialization. However, it should be pointed out that we also look forward to its application in building, industrial, and other aspects.

**Figure 1:**
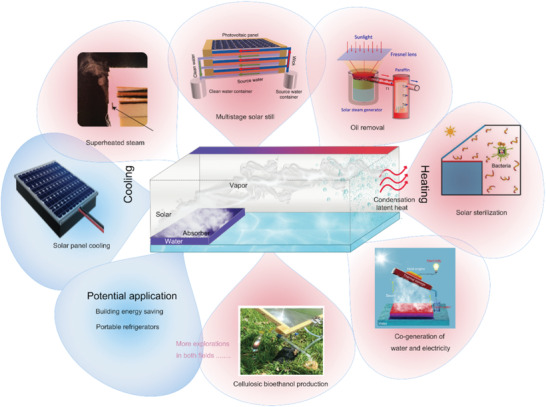
Schematic illustration of the interfacial solar steam/vapor generation for heating and cooling.

Thanks to the improvement of solar to steam/vapor conversion efficiency, interfacial solar steam/vapor generation has achieved significant progress in heating and cooling in the past few years. For example, in heating (refers to the utilization of latent heat of steam/vapor condensation), efficient solar steam generation has been achieved under lower optical concentrations, even 1 Sun, mainly through absorber and device's optical and thermal regulation. It has been applied to cogeneration of water and electricity,^[^ ^64^ ^]^ multistage solar still,^[^ ^65^, ^66^, ^67^, ^68^, ^69^, ^70^, ^71^, ^72^ ^]^ sterilization,^[^ ^73^, ^74^, ^75^, ^76^, ^77^ ^]^ superheated steam,^[^ ^78^, ^79^ ^]^ etc.^[^ ^80^, ^81^, ^82^ ^]^ (as shown in **Figure** **1**, right). Cooling,^[^ ^83^, ^84^, ^85^, ^86^, ^87^, ^88^, ^89^, ^90^ ^]^ even subambient cooling,^[^ ^91^, ^92^, ^93^, ^94^, ^95^ ^]^ was also achieved through the morphology and structure of the absorber and device designs and applied to the cooling of solar panels (Figure 1, left). However, this direction is still in the infant stage and has many fundamental and real application challenges. Although in the past few years, many impressive review papers have been published about interfacial solar steam/vapor generation, most of them are focusing on how to improve further the water output of the device and the durability of the absorber and pay less attention to the heating and cooling in solar to steam/vapor conversion process. Based on this background, this review innovatively introduces and discusses the interfacial solar steam/vapor generation working principle to achieve heating and cooling, state‐of‐the‐art progress, opportunities, and challenges faced. We believe it will enlighten the next research step and further promote the rapid development and application of interfacial solar steam/vapor technology in heating and cooling.

## 2 Interfacial Solar Steam/Vapor Generation for Heating

**Figure 2:**
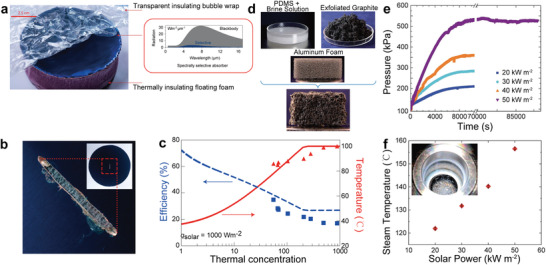
a) A photograph of the thermal concentration system composed of the bubble wrap, commercial selective absorber, and thermal foam insulates. The inset compares thermal radiative losses at 100 °C from selective absorber and blackbody. b) Evaporation slot. The inside of the evaporation slot is black water supply fabric. The inset shows where the evaporation slot is cut. c) Solar to steam conversion efficiency and steam temperature over thermal concentration. a–c) Reproduced with permission.^[^ ^62^ ^]^ Copyright 2016, Nature Publishing Group. d) Flow chart of the absorber's preparation, which includes PDMS + brine solution, exfoliated graphite, and aluminum foam. e) The steam pressure over time and solar concentration. f) The corresponding steam temperature over solar concentration. The inset shows the fluid at the surface of the absorber is at the boiling state. d–f) Reproduced with permission.^[^ ^63^ ^]^ Copyright 2016, Royal Society of Chemistry.

Freshwater, electricity, sterilization, etc., have become indispensable elements of our lives. However, in some remote areas where the infrastructure is lacking, freshwater, electricity, sterilization, etc., are still not well available. Interfacial solar steam generation technology uses relatively abundant solar energy and water for heating, which is expected to provide a competitive solution for obtaining fresh water, electricity, sterilization, etc., in remote areas. The heating realization is through solar energy to heat the absorber to generate high‐temperature steam/vapor, which quickly diffuses to the heated area to release the latent heat of condensation. Typically, the interfacial solar vapor temperature is about 40 °C under 1 Sun. Even though this temperature is not high, it can still be applied to multistage solar by releasing the latent heat of condensation to heat the next stage to evaporate (see **Table** **1**). The high‐temperature steam (≥100 °C) is usually obtained in two ways, 1) thermal concentration and heat localization. The high‐temperature steam is achieved by conducting the total absorbed heat of the absorber into a smaller area of evaporation. As shown in **Figure** **2**a,b, Ni et al.^[^ ^62^ ^]^ developed a thermal concentration system composed of transparent insulating bubble wrap, selective absorber, thermally insulating floating foam, and water supply path, which can generate ≈100 °C steam with ≈20% solar to steam conversion efficiency under 1 Sun (Figure 2c) through a three‐step design. The first step is to choose a selective absorber to achieve higher optical absorption *α* (*α* = 0.93) and lower thermal emittance *ε* (*ε* = 0.07). The second step is to conduct thermal insulation treatment using a transparent bubble wrap on the upper and a polystyrene foam disk on the bottom to reduce convection loss to ambient and conductive and radiative heat losses to the water underneath. The third step is to use thermal concentration (*C*_therm_ = 1300×, *C*_therm_, the ratio of the total absorbed area to the evaporation area) (Figure 2b). 2) Optical concentration. High‐temperature steam can also be obtained by increasing the energy density by optical concentration. Sajadi et al.^[^ ^63^ ^]^ reported a flexible artificially networked material composed of polydimethylsiloxane/brine solution, exfoliated graphite, and the scarifying aluminum foam for high‐temperature steam (Figure 2d). The result showed that it could generate 100–156 °C of steam at the pressure range of 100–525 kPa under the light intensity of 20–50 kW m^−2^ in a closed system (Figure 2e,f). Compared with optical concentration, thermal concentration has significant advantages in cost and convenience. However, the high‐temperature steam efficiency generated by thermal concentration is still low. The high‐temperature steam was used for sterilization, cogeneration of water and electricity, superheated steam, etc. (see Table 1).

**Table 1:** Summary of interfacial solar steam/vapor generation for heating

Heating	Materials and/or structures	Steam/vapor temperature	Performances	References
Multistage solar still (ten‐stage)	TiNOx‐a commercial selective absorber (*α* = 0.95, *ɛ* = 0.04)	≈60 °C	3 L m^−2^ h^−1^ water production from seawater was achieved under laboratory conditions.	[65]
Multistage solar still (three‐stage)	Solar cell (*α* = 0.92)	61.8 °C	>1.64 kg m^−2^ h^−1^ water output from seawater and >11% electricity generation were achieved.	[66]
Multistage solar still (three‐stage)	Solar cell (*α* = ≈0.80)	≈40 °C	The system outputted fresh water with a rate of 1.11 kg m^−2^ h^−1^ and 66.6 W m^−2^ electricity power.	[68]
Multistage solar still (ten‐stage)	Solar absorber (*α* = 0.93)	≈70 °C	385% solar to vapor conversion efficiency with a water evaporation rate of 5.78 L m^−2^ h was achieved under 1 Sun.	[69]
Multistage solar still (five‐stage)	Solar cell (*α* = ≈0.83)	≈60 °C	The device outputted 1.17 kg m^−2^ h^−1^ fresh water, 97 W m^−2^ electricity, and 1.02 kg m^−2^ per day salt collection.	[70]
Multistage solar still (two‐stage)	Commercial spectrally selective absorber (Bluetec, Germen) (*α* = 0.95, *ɛ* = 0.05)	45–50 °C	The device achieves water productivity of 1.02 kg m^−2^ h^−1^ with a solar to vapor conversion efficiency of 72% under 1 Sun.	[71]
Solar sterilization	Au nanoshells dispersions	≈140 °C	The device can achieve a steam temperature of 132 °C in a volume of 14.2 L, which can reach Food and Drug Administration sterilization requirements.	[73]
Solar sterilization	rGO/PTFE composite membrane (*α* = ≈0.90)	>140 °C	>120 °C steam was obtained; the sterilization capability was successfully demonstrated by both biological and chemical sterilization tests.	[74]
Solar sterilization	Biochar‐materials (*α* = ≈0.96)	132 °C	A full sterilization cycle within 8.4 min and ≈99.999999% inactivation of the pathogen can be achieved.	[76]
Solar sterilization	Selective absorber (*α* > 90%, *ɛ* < 10%)	128 °C	The steam at 128 °C and 250 kPa was achieved that is sufficient for finishing the medical sterilization cycle.	[77]
Superheated steam	Selective absorber (TiNOx) (*α* = 0.93, *ɛ* = 0.07)	165 °C	121 °C‐superheated steam can be achieved with an averaged solar flux of ≈600 W m^−2^.	[75]
Superheated steam	Selective absorber	133 °C	>130 °C superheated steam can be achieved under 1 Sun.	[79]
Cogeneration of clean water and electricity	Graphite/Nonwoven (*α* = ≈0.98)	146 °C	While achieving a solar to steam conversion efficiency of 72.2%, an electricity power of 1.23% efficiency can be achieved.	[64]
Cogeneration of clean water and electricity	Nitrogen enriched carbon sponge (*α* = ≈0.96)	≈47 °C	The system can achieve 90% solar to vapor conversion efficiency and 240.7 mW m^−2^ electricity production.	[82]
Other (Production of cellulosic bioethanol)	Au nanoshells dispersions	≈160 °C	The sugars were successfully obtained by steam treatment; the ethanol was also purified by solar steam distillation.	[80]
Others (Removal of paraffin deposits)	Selective absorber (TiNOx) (*α* = 0.95, *ɛ* = 0.05)	100 °C	48% solar to steam conversion efficiency can be achieved; the paraffin in the tube was effectively removed.	[81]

## 3 Multistage Solar Still

**Figure 3:**
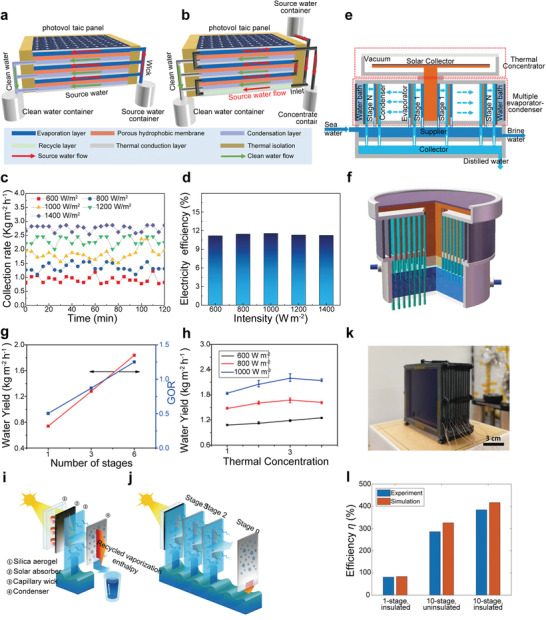
Schematic illustration of the integrated photovoltaics‐membrane distillation devices. Operating in a a) dead‐end mode and b) cross‐flow mode. c) Collection rate and d) electricity generation efficiency under the different solar concentrations of the integrated photovoltaics‐membrane distillation devices with dead‐end mode. a–d) Reproduced with permission.^[^ ^66^ ^]^ Copyright 2019, Nature Publishing Group. e) Schematics and working principle of the multistage distiller with thermal concentration. f) 3D schematic of a six‐stage thermal concentrated distiller. g) Water yield and gain output ratio of the distiller over different stages without thermal concentration. h) Specific water yield of the distiller with six‐stage over thermal concentration and solar power density. e–h) Reproduced with permission.^[^ ^68^ ^]^ Copyright 2020, Elsevier. i) Key components (including a silica aerogel, a solar absorber, a capillary wick, and a condenser) in the first stage of the multistage distiller. j) Schematic diagram of the working principle of multistage distillation. k) Optical image of the multistage distiller. l) Solar to vapor conversion efficiency of ten‐stage distiller with thermal insulation compared with a one‐stage and a ten‐stage device without thermal insulation. i–l) Reproduced with permission.^[^ ^69^ ^]^ Copyright 2020, Royal Society of Chemistry.

For solar steam/vapor generation, even if a conversion efficiency of 100% is achieved, the theoretical single‐stage water production rate is about 1.47 kg m^−2^ h^−1^ under 1 Sun (assuming the phase change enthalpy of water is not changed by the material).^[^ ^95^ ^]^ To further increase water production, recycling the condensation latent heat to form multistage is effective.^[^ ^65^, ^66^, ^67^, ^68^, ^69^, ^70^, ^71^, ^72^ ^]^ As shown in **Figure** **3**a,b, Wang et al.^[^ ^66^ ^]^ chose commercial polycrystalline silicon as the absorber (*α* > 92%) and combine a three‐stage membrane distillation device on the back of the solar panel to directly utilize its waste heat to drive water distillation (Figure 3a,b). The result showed that the water production rate could reach 1.79 kg m^−2^ h^−1^ under 1 Sun (Figure 3c). Additionally, the solar panel can generate electricity with energy efficiency >11% simultaneously (Figure 3d). From the perspective of solar to heat conversion, the solar panel is not an ideal absorber, reducing the device's water production. However, the advantage of this strategy is to improve the overall efficiency of the device and obtain additional power. Alternatively, increasing optical absorption and thermal insulation is beneficial to increase the device's water production. Huang et al.^[^ ^68^ ^]^ selected a more efficient selective absorber with *α* = 0.935 and *ɛ* = 0.150 to improve solar to heat conversion efficiency (Figure 3e,f). Moreover, the concentric expansion evaporator–condenser structure was designed to suppress the heat losses and water diffusion resistance. The result showed that the water production rate of this six‐stage distillation device with three times thermal concentration could reach 2.2 kg m^−2^ h^−1^ under 1 Sun (Figure 3g,h), and the water production could reach 3.9 kg m^−2^ per day even at a low solar power intensity of 415 W m^−2^ in the outdoor. To further improve the water output, Xu et al.^[^ ^69^ ^]^ analyzed heat and mass transport mechanisms in multistage solar still and designed a ten‐stage distillation device (Figure 3i–k). As shown in Figure 3i, the first stage includes a transparent silica aerogel layer (Transmittance = 0.95) cover on a solar absorber (*α* = 0.93) to decrease the heat losses from the absorber to ambient, a capillary wick, and a condenser. Each of the following stages consists of a capillary wick and a condenser separated by an air gap. After structural optimizations, the result showed that more than 75% of vaporized water was collected through condensation, and 5.78 L m^−2^ h^−1^ water output rate (solar to vapor conversion efficiency of 385%, Figure 3l) could be achieved under 1 Sun.

Apart from these impressive experimental results, Zhang et al.^[^ ^72^ ^]^ theoretically discussed heat and mass transfer mechanisms and the potential optimized direction of the multistage solar still. Specifically, they analyzed the impact of the number of stages, sidewall insulation, and geometric configuration of the device on the final efficiency. The simulation results show that 1) there is an upper limit for increasing the water production by adding stages (the first several stages contribute the most to vapor generation) determined by the device's geometric configuration. 2) The thermal insulation of the sidewall is critical that can lead to an order‐of‐magnitude enhancement in conversion efficiency by reducing heat loss. 3) For a fixed device width, constrained by the wicking length, the highest efficiency of the device can be achieved by optimizing the device width. For example, the theoretical prediction results showed that for a 15 cm wide device with excellent thermal insulation, the device's efficiency could exceed 700% when the unit stage thickness is equal to 0.25 cm. It can be found that there is still a significant gap between the highest achievable efficiency and the theoretical efficiency. This theoretical work is expected to provide clear guidance for the subsequent optimization and design of passive desalination technologies.

## 4 Solar Sterilization

Solar steam sterilization is a competitive technology for public health, especially in areas lacking infrastructures. Typically, the steam temperature needs to reach 121 °C for 15 min or 132 °C for 5 min to effectively achieve sterilization (note, the steam temperature refers to the saturated steam temperature rather than the superheated steam temperature).^[^ ^73^ ^]^ Interfacial solar steam generation with a high evaporation rate has brought new opportunities and breakthroughs for this technology.^[^ ^74^, ^75^, ^76^, ^77^ ^]^ Li et al.^[^ ^76^ ^]^ compared the response time and energy consumption of traditional volumetric and interfacial heating in detail through an isothermal model with the fixed solar power density and thermodynamic assumptions. The conclusion of the model can be expressed by Equation (1) (the corresponding derivation process refers to ref. [76]).

(1) $$\frac{\Delta E_{H,V,\mathrm{Ts}}}{\Delta E_{H,I,\mathrm{Ts}}}=\frac{\tau_{H,V,\mathrm{Ts}}}{\tau_{H,I,\mathrm{Ts}}}\;\approx 1+148\frac{V_{W}}{V_{S}}$$

where ∆*E*_H,V,Ts_ and ∆*E*_H,I,Ts_ represent the input energy for volumetric and interfacial heating systems from initial state to *Ts* (the typical experimental temperature is *Ts* = 121 °C for this experiment); *τ*_H,V,Ts_ and *τ*_H,I,Ts_ are the time interval of heating phase to *Ts* of volumetric and interfacial heating. *V*_W_ and *V*_S_ are water volume and steam volume. It is clear that compared with volumetric heating, interfacial heating has apparent advantages in terms of energy consumption and response time. For example, for a solar autoclave with *V*_W_/*V*_S_ = 1, the solar autoclave's energy consumption and impact time using interfacial heating is 149 times smaller than volumetric heating.

**Figure 4:**
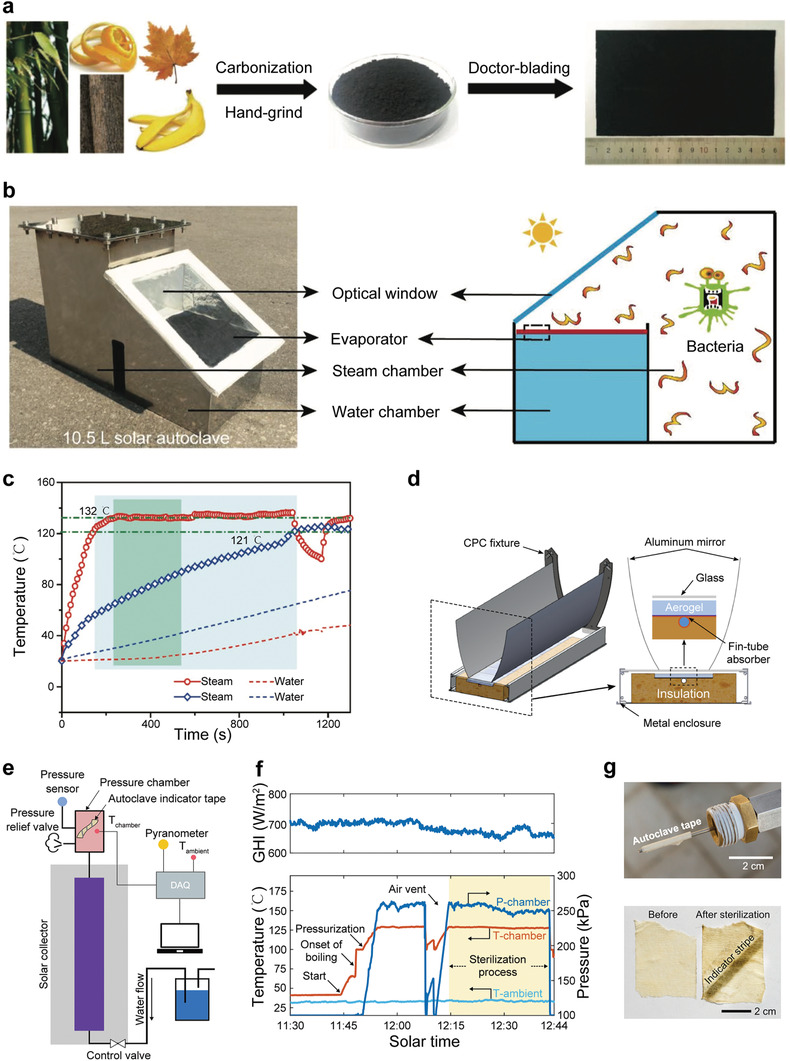
a) Preparation flow chart of biochar‐based solar absorbers. b) A photograph and a schematic of the interfacial heating‐based solar autoclave with the volume of 10.5 L. c) Steam and water temperature of interfacial and nanofluid (volumetric heating) over time. The insert blue and green boxes indicate the sterile regime (121 °C for 15 min or 132 °C for 5 min). a–c) Reproduced with permission.^[^ ^76^ ^]^ Copyright 2018, Wiley‐VCH. d) The structure schematic of the assembled solar autoclave. e) Schematic of the device for sterilization. A pressure chamber was connected to the solar steam generator. f) Chamber pressure, temperature, and solar power density during the experiment. The insert of yellow shaded area indicates the effective autoclaving period. g) The changes of autoclave indicator tape before and after the sterilization process. d–g) Reproduced with permission.^[^ ^77^ ^]^ Copyright 2020, Cell Press.

Guided by theoretical analysis, Li et al.^[^ ^76^ ^]^ prepared efficient absorbers (*α* = 96%) by carbonizing some biomass materials (**Figure** **4**a), and performed a systematic study on interfacial solar steam‐based sterilization (Figure 4b). As shown in Figure 4c, the steam temperature of interfacial heating can quickly reach 132 °C (<5 min), but for volumetric heating, the steam temperature only reaches 121 °C in 15 min. The sterilization results showed ≈99.999999% sterilization efficiency for all the target bacteria could be achieved. Unlike the above method of concentrating light to obtain high‐temperature steam, Zhao et al.^[^ ^77^ ^]^ adopted thermal concentration strategy with deliberate optical and thermal design to achieve high‐temperature steam (Figure 4d). In this experiment, silica aerogel with high transparency (Transmittance = 0.95) and low thermal conductivity was selected to cover on a selective absorber to suppress the heat loss without significantly reducing the input of sunlight. Moreover, a no compound parabolic concentrator (CPC) with a geometric concentration ratio of 2.133 (acceptance angle = 25°) was used to increase light intensity. As a result, 100 °C saturated steam with 56% (no CPC) and 47% (with CPC) efficiency under realistic weather conditions can be realized. When this device is connected to a pressure chamber (Figure 4e), the prototype can continuously deliver pressurized steam at 128 °C and 250 kPa, which can be effectively used for sterilization (Figure 4f,g).

These devices are very close to practical. In the follow‐up research, higher performance absorbers and insulation are desired to obtain better performance. More importantly, the cost of materials should be further reduced. For example, it is meaningful to explore large‐scale, low‐cost preparation of low thermal conductivity, high‐transparent thermal insulation materials, like silica aerogel.

## 5 Cogeneration of Water and Electricity

Clean water and electricity are essential elements for our daily lives. Unfortunately, many areas lack the necessary clean water and electricity, especially in some underdeveloped and remote areas. In these areas, interfacial solar water purification is a promising technology to alleviate the clean water and electricity crisis by cogeneration of clean water and electricity.^[^ ^64^ ^]^ As mentioned before, interfacial solar heating energy is localized at the floated absorber. Thus, there will be a temperature difference between absorber and ambient, which will bring corresponding heat conduction, heat convection, and heat radiation losses. Moreover, the latent heat of steam condensation is usually lost to the ambient. These limit the entire conversion efficiency of the device. Interestingly, electricity generation can be introduced into solar steam/vapor generation systems to utilize these heat losses to realize cogeneration of water and electricity. So far, some power generation technologies are used in conjunction with the interfacial solar steam/vapor generation to achieve cogeneration of clean water and electricity.^[^ ^64^, ^82^, ^96^, ^97^, ^98^, ^99^, ^100^, ^101^, ^102^, ^103^, ^104^, ^105^, ^106^, ^107^, ^108^ ^]^

**Figure 5:**
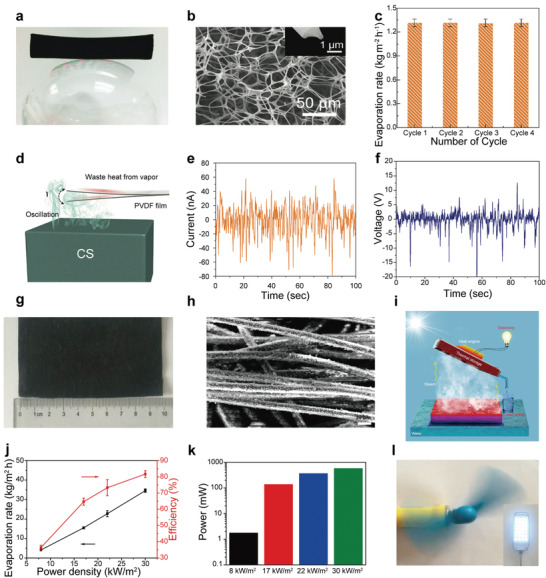
a) Photograph of a nitrogen‐enriched carbon sponge standing on the bubble balloon. b) Scanning electron microscope (SEM) images of nitrogen‐enriched carbon sponge. The inset is the cross‐sectional image of carbon sponge fibrils. c) The evaporation of carbon sponge over the cycle. d) Schematic of vapor generation‐induced electric potential by carbon sponge. The e) output currents and f) voltages of the PVDF films over time during the evaporation process. a–d) Reproduced with permission.^[^ ^82^ ^]^ Copyright 2018, Wiley‐VCH. g) Optical image and h) SEM image of graphite/nonwoven absorber. i) Schematic of cogeneration of clean water and electricity based on interfacial solar steam enthalpy recycling. j) The evaporation rate and solar to steam conversion efficiency over solar power density. k) Maximum output power of the thermoelectric device over solar power density. l) Optical image of an operating electric fan and light‐emitting diode driven by thermoelectric device. g–l) Reproduced with permission.^[^ ^64^ ^]^ Copyright 2018, Cell Press.

Among them, a more competitive strategy is to use the latent heat of steam condensation to generate electricity. Zhu et al.^[^ ^82^ ^]^ developed a high efficient solar absorber, nitrogen‐enriched carbon sponge (**Figure** **5**a,b), for solar vapor generation. The result showed that a 1.39 kg m^−2^ h evaporation rate could be achieved (Figure 5c). Moreover, they used a ferroelectric fluoropolymer polyvinylidene fluoride (PVDF) to harvest the thermomechanical responses from the generated vapor to achieve power generation (Figure 5d). The coupling of the pyroelectric and piezoelectric effects is achieved via heating–cooling and slight oscillation of PVDF film during the solar to vapor conversion process. The results showed that the system could reach 90% solar to vapor conversion efficiency and the output power of 240.7 mW m^−2^ (Figure 5e,f). To increase the steam temperature to increase the power generation, Li et al.^[^ ^64^ ^]^ prepared an efficient graphite/nonwoven absorber (Figure 5g,h), raised the steam temperature by concentrating light, and finally used steam to drive a thermoelectric module to generate electricity (Figure 5i). The results showed that while obtaining a solar to steam conversion efficiency of 72.2% (81.7% in a semiclosed system, as shown in Figure 5j), a power generation efficiency of 1.23% could be obtained (Figure 5k), which could be supported by continuous operation of an electric fan with 1 W and 28 light‐emitting diodes with a total power of 1.5 W (Figure 5l).

At present, whether it is using steam to drive pyroelectric‐piezoelectric or thermoelectric, the power generation efficiency is still too low to meet actual demands. Li et al.^[^ ^64^ ^]^ gave a theoretical analysis to improve further the performance of cogeneration of clean water and electricity based on the thermoelectric module. For a thermoelectric module, its efficiency (*η*) can be expressed by Equation (2).^[^ ^64^ ^]^

(2) $$\eta \;=\frac{\Delta T}{T_{h}}\;\frac{\sqrt{1+ZT_{\mathrm{avg}}}-1}{\sqrt{1+ZT_{\mathrm{avg}}}+\frac{T_{c}}{T_{h}}}$$

where ∆*T* is the temperature difference between the cold and hot sides. *ZT*_avg_ is the thermoelectric figure of merit. *T*_c_ and *T*_h_ are the thermoelectric cold side and hot side temperatures. It can be found that if the steam temperature can be further increased, the corresponding power generation efficiency will also increase significantly. For example, when the steam temperature can be raised to 400 K, and the thermoelectric module with *ZT* = 2 is used for power generation, the corresponding solar to steam and electricity conversion efficiency can reach ≈95% and 7.9%.

## 6 Interfacial Solar Steam/Vapor Generation for Cooling and Subambient Cooling

Evaporative cooling is a type of passive cooling technology, which achieves cooling by taking away energy through water evaporation.^[^ ^83^, ^84^ ^]^ It is pervasive in our lives and used to cool ambient and suppress dust on yards, roads, etc. It is also widely used in industries, for example, cooling the condensers in thermal power plants. The development of interfacial solar steam/vapor generation has dramatically increased the evaporation rate, bringing new changes for evaporation cooling development. Here, we mainly discuss the combination of evaporation cooling and interfacial solar steam/vapor in two situations: one is cooling objects above room temperature (i.e., cooling), the other is working below room temperature (i.e., subambient cooling) (see **Table** **2**). These are two new directions, especially for subambient cooling. As shown in Table 2, it can be found that although the subambient phenomenon has been realized, the attention is still on water production. We believe that interfacial solar vapor generation for subambient cooling is an exciting field and hope that the research community can pay attention to this phenomenon.

**Table 2:** Summary of interfacial solar vapor generation for cooling

	Materials and/or structures	Cooling power	Performances	References
Cooling	PAM‐CNT‐CaCl_2_ hydrogel	295 W m^−2^	It can reduce solar cells by at least 10 °C in laboratory testing. Outdoor (Saudi Arabia) test results show that the power generation of solar panels in the summer and winter can be increased by 19% and 13%.	[85]
Cooling	Li‐PAAm hydrogel	—	The temperature of solar cells can be reduced by 17 °C under 1 Sun, and the efficiency of polycrystalline silicon solar cells can be increased from 14.5% to 15.5%.	[86]
Cooling	Combined with a five‐stage distillation device	—	The temperature of the solar cell can be lowered by 15 °C under 1 Sun, which leads to an 8% increase in its electricity generation.	[87]
Cooling	Combined with a five‐stage distillation device	—	The efficiency of solar cells can be increased by 1%.	[90]
Cooling	Combined with a one‐stage distillation device	—	The efficiency of electricity generation can be enhanced by 7.9% under one sun.	[100]
Cooling	MIL‐101(Cr)	—	8.6 °C temperature drop can be obtained for 25 min at a heating power of 1.5 W.	[109]
Subambient cooling	Cotton cores wrapped with plant cellulose	—	≈4 °C temperature drop can be achieved in the side surface under 1 Sun.	[95]
Subambient cooling	3D interconnected porous carbon foam	—	≈3 °C temperature drop can be achieved in the side surface under 1 Sun.	[91]
Subambient cooling	3D cylindrical cup‐shaped structures of mixed metal oxide	—	≈3 °C temperature drop can be achieved in the outer wall under 1 Sun.	[92]
Subambient cooling	Triangle structure with *θ* of 22.4°.	—	≈6 °C temperature drop can be achieved in the side surface under 1 Sun.	[93]
Subambient cooling	Heatsink‐like evaporator composed of porous nanocarbon composites	—	≈11 °C temperature drop can be achieved in the structure center under 1 Sun.	[94]

Note: Temperature drop refers to a temperature lower than room temperature.

## 7 Cooling

**Figure 6:**
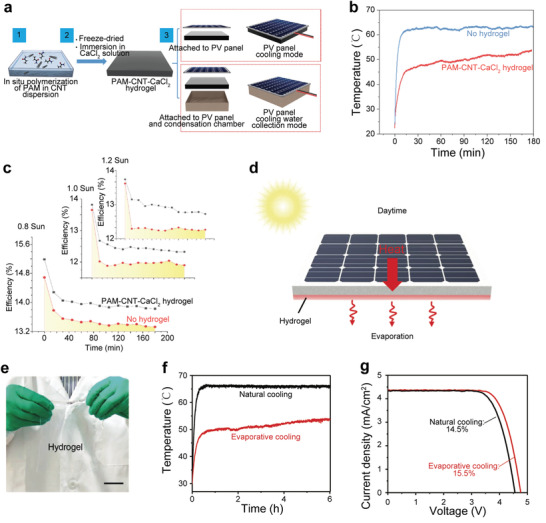
a) Schematic of the PAM‐CNT‐CaCl_2_ synthesis process (steps 1 and 2) and two modes of operation combined with solar panel (step 3). b) The temperature change of the solar panel with and without the PAM‐CNT‐CaCl_2_ hydrogel cooling layer under 1 Sun. c) Parallel comparison of solar panel efficiency with and without the cooling layer attached. a–c) Reproduced with permission.^[^ ^85^ ^]^ Copyright 2020, Nature Publishing Group. d) Photograph of the Li‐PAAm hydrogel. e) Schematic of the working principle of the Li‐PAAm hydrogel to cool the silicon solar cell. f) Surface temperature and g) photocurrent density over voltage of the solar cell with and without evaporative cooling under 1 Sun. d–f) Reproduced with permission.^[^ ^86^ ^]^ Copyright 2020, Wiley‐VCH.

As mentioned above, solar vapor generation is a cooling process, and thus it can maintain the device to work at low temperature under sunlight. It is significant for devices whose conversion efficiency and service life are sensitive to high temperatures. For solar panels, the conversion efficiency will decrease by 0.4–0.5% for each degree of temperature increase. Additionally, the high temperature shortens the lifetime of the solar panel.^[^ ^85^ ^]^ Thus, effective cooling strategies of the solar panel are highly desired for efficient and long‐term power. An effective strategy to address this challenge is the combination of solar panels and adsorbent, which is coated on the back of the solar panel to achieve cooling. The working principle is that the adsorbent can absorb and store the water at night. During the day, solar panels are heated under solar irradiation, and water molecules will be evaporated and quickly dissipate the waste heat into the ambient to achieve cooling. At night, the adsorbent will re‐absorb water to gain circulation. As shown in **Figure** **6**a, Li et al.^[^ ^85^ ^]^ prepared a new type of sorbent, carbon nanotube (CNT)‐embedded cross‐linked polyacrylamide (PAM) as substrate and calcium chloride as water vapor sorbent (PAM‐CNT‐CaCl_2_), which can extract and store a lot of water from the air, even under relative humidity (RH) < 35%. As a result, the PAM‐CNT‐CaCl_2_ can provide an average cooling power of 295 W m^−2^ under 1 Sun for solar panels and achieve cooling of more than 10 °C (Figure 6b). As shown in Figure 6c, the efficiency of the solar panel with PAM‐CNT‐CaCl_2_ is 0.8% higher than that without PAM‐CNT‐CaCl_2_. Outdoor test results showed that this technology could increase the power generation of solar panels by ≈19% and ≈13% in the summer and winter. Pu et al.^[^ ^86^ ^]^ used a thin layer of lithium‐ and bromine‐enriched polyacrylamide (Li‐PAAm) hydrogel to achieve the same function through combing the hydrogel on the back of the solar panel because Li‐PAAm can also be reversibly absorbed and released air water molecules to achieve solar panel cooling (Figure 6d,e). The result showed that the temperature of solar panels covered with Li‐PAAm rose from 49 to 53 °C after 6 h operation. Under the same conditions, the bare solar panels were stable at 66 °C (Figure 6f). The efficiency of the solar panels was increased from 14.5% to 15.5% (Figure 6g). Outdoor experiments (RH = 28%, temperature ranging from 28 to 35 °C, solar intensity ≈0.7 kW m^−2^) showed that the technology could increase the efficiency of solar panels by 15.6%.

This technology shows a strong application prospect because it has the following advantages: 1) it has a simple technical design, 2) it is not restricted by the availability of liquid water, 3) it does not require external energy input, and 4) it can be further realized the cogeneration of clean water and electricity.^[^ ^85^ ^]^ To further improve the device's performance, Li et al. analyzed the current bottleneck and prospects of the technology. Specifically, they systematically established the driving force of water vapor sorption and desorption, mass transfer, and heat transfer models to explore potential optimization directions. The result shows that: 1) The development of some new sorbent materials with larger sorption windows and lower desorption temperature improves cooling performance. 2) In terms of mass transfer, the current significant bottleneck lies in the transfer of water molecules in sorption material, which often limits the maximum water uptake capacity during nighttime. Therefore, developing some new sorbents with superior water transport is also critical. 3) The inspiration from the thermal model is that increasing the thermal conductivity and emissivity of the sorption material is beneficial to enhance the cooling performance of the sorption material. These have vital guiding significance for developing this technology in the future. In addition to the above discussion, the materials' corrosiveness, cost, and durability should also be considered as important material evaluation indicators.

**Figure 7:**
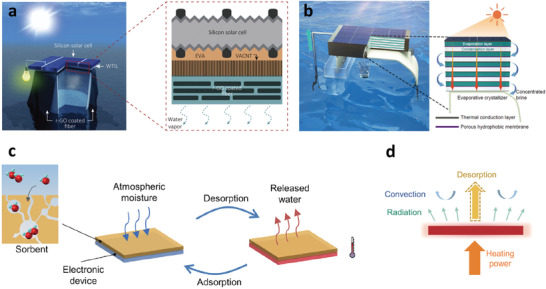
a) Schematic and device structure of the integrated solar panel and solar still. Reproduced with permission.^[^ ^100^ ^]^ Copyright 2020, Cell Press. b) Schematic and device structure of the integrated solar panel and five‐stage solar still. Reproduced with permission.^[^ ^87^ ^]^ Copyright 2021, Cell Press. c) Schematic diagram of thermal management of electronic devices with the sorption–desorption process. d) Heat transfer diagram of an electronic device with the desorption process. c,d) Reproduced with permission.^[^ ^109^ ^]^ Copyright 2020, Cell Press.

Different from the above method that combines solar panels with adsorbents, another strategy is to connect solar panels to solar still. Since the back of the solar panels is connected to an evaporator, evaporation cooling can also be achieved.^[^ ^87^, ^100^ ^]^ As shown in **Figure** **7**a, Xu et al.^[^ ^100^ ^]^ connected polysilicon solar panels to the solar still, and enhanced the thermal conduction between the solar panels and the solar still by designing an interface thermal conductive material (vertically aligned CNTs‐embedded ethylene‐vinyl acetate member) with thermal conductivity of ≈1 W mK^−1^. The results show that a prototype hybrid tandem solar device can increase the power generation of solar panels by 7.9% and obtain 0.80 kg m^−2^ h^−1^ of freshwater under natural sunlight. To improve the total efficiency of the device, Wang et al.^[^ ^87^ ^]^ connected the solar panels with five‐stage solar still to improve water output (Figure 7b). This system can reduce the solar panels' temperature by 15 °C and increase the power generation by 8% for bare solar panels. Moreover, the five‐stage solar still can produce 2.45 kg m^−2^ h^−1^ of freshwater. These are up‐and‐coming technologies. It is expected that the devices with higher performance and practicability will be designed in the future.

In addition to the great potential of evaporative cooling on the solar panels discussed above, it also has shown excellent application prospects in the thermal management of electronic devices.^[^ ^86^, ^109^ ^]^ Recently, Wang et al.^[^ ^109^ ^]^ successfully applied sweat cooling with the sorption–desorption process to the thermal management of electronic devices (Figure 7c,d). Specifically, they discussed the performance of different sorbents under different RH conditions through simulations and found MIL‐101(Cr) that performed better under the ambient condition of RH 40%. The result showed that a maximum temperature drop of 8.6 °C for 25 min at a heating power of 1.5 W could be achieved by coating 0.288 g of MIL‐101(Cr) powders on the surface of the device. Also, they gave clear guidelines on the future optimization direction. 1) The sorbents should be climate‐specific. 2) The sorbents with linear water uptakes over RH are desired. 3) Except for considering high uptake capacities, it is also crucial to explore the kinetics mechanism to design more reasonable geometric and morphological factors.

## 8 Subambient Cooling

Like cooling, subambient cooling also plays a vital role in energy‐saving and food and medicine storage, where thermal management at subambient temperatures is necessary.^[^ ^84^, ^110^, ^111^, ^112^, ^113^, ^114^, ^115^, ^116^, ^117^, ^118^, ^119^, ^120^ ^]^ However, most subambient cooling technologies still rely on electrical energy. In the passive cooling technology (without electricity), evaporative cooling and radiative cooling (radiative cooling is to radiate its energy to the cold outer space [3 K] through the atmospheric window [8–13 µm]^[^ ^110^, ^111^, ^112^, ^113^, ^114^, ^115^, ^116^, ^117^, ^118^, ^119^, ^120^ ^]^) are desirable technologies. Currently, Aili et al.^[^ ^83^ ^]^ systematically analyzed evaporative cooling through theoretical calculations and compared it with radiative cooling in detail. The results show that evaporative cooling is better suited for high‐temperature and low‐humidity weather conditions. For example, at RH = 13% and an ambient temperature of 26 °C, the subambient temperatures of the radiative and evaporative coolers are −13.5 and −15.0 °C. From the perspective of evaporation kinetics, high temperature and low humidity are more conducive to evaporation. However, radiative cooling is a relatively weaker dependence on temperature and humidity. It can be found that evaporation cooling has its advantages in subambient cooling, showing a desirable application prospect.

**Figure 8:**
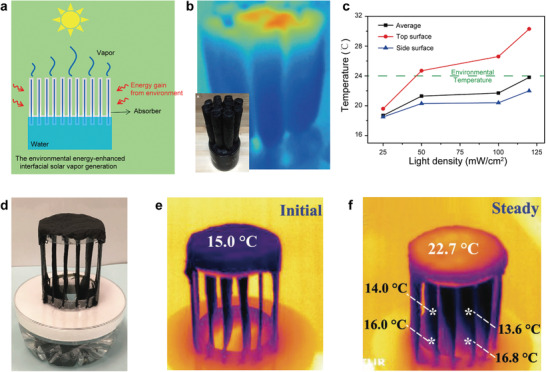
a) Schematic of environmental energy‐enhanced interfacial solar vapor generator. b) Infrared images (IR) and photograph (insert) of the environmental energy‐enhanced interfacial solar vapor generator under 1 Sun. c) Top and side surface temperatures of the environmental energy‐enhanced interfacial solar vapor generator over solar power density. a–c) Reproduced with permission.^[^ ^95^ ^]^ Copyright 2018, Cell Press. d) Photographs of the test setup. e) Initial and f) steady IR images of the six‐fin heat sink‐like evaporator under 1 Sun after 30 min. d‐f) Reproduced under the terms of the Creative Commons CC‐BY license.^[^ ^94^ ^]^ Copyright 2021, The Authors. Published by Wiley‐VCH.

As mentioned before, assuming the phase change enthalpy of water is not changed by the materials, the theoretical single‐stage water production is about 1.47 kg m^−2^ h^−1^ under 1 Sun. In addition to using multistage to improve water production, another strategy is introducing environmental energy to enhance evaporation. However, a prerequisite for this strategy is that the evaporation temperature of the absorber should be lower than the ambient temperature. In other words, if we achieve the environmental energy‐enhanced evaporation, subambient cooling is performed simultaneously.^[^ ^88^, ^89^, ^90^, ^91^, ^92^, ^93^, ^94^ ^]^ For daytime radiative cooling, in addition to radiating its energy to outer space through infrared radiation, daytime radiative cooling also needs to reflect solar energy (mostly >95%). Unlike daytime radiative cooling's reflection, currently, evaporation cooling uses the height of the absorber to suppress the downward input of solar energy through evaporation at the upper part of the absorber and realizes subambient cooling on the sidewall or the lower part. As shown in **Figure** **8**a, Li et al.^[^ ^95^ ^]^ demonstrated environmental energy‐enhanced cylindrical vapor generator, composed of cotton cores wrapped with plant cellulose, sufficient water supply, high absorption, high evaporation area, and a low projection ratio. The results showed that the structure could achieve a 1.62 kg m^−2^ h^−1^ evaporation rate under 1 Sun. More exciting, the sidewall temperature of the structure is still about 4 °C lower than room temperature under 1 Sun irradiation (Figure 8b,c). Similar phenomena have been observed in 3D cylindrical cup‐shaped and triangle structures with different apex angles.^[^ ^92^, ^93^ ^]^ To further improve the water evaporation rate, Wu et al.^[^ ^92^ ^]^ developed a six‐fin photothermal heat sink‐like evaporator composed of porous nanocarbon composites (Figure 8d). The result showed that this structure achieved a 4.02 kg m^−2^ h^−1^ evaporation rate and a maximum 11 °C temperature drop in the center area under 1 Sun (Figure 8e,f). In the research of environmental energy‐enhanced evaporation, in addition to thinking from the perspective of increasing water production, using this strategy to explore subambient cooling is also an exciting direction (more discussion can be found in the outlook part).

## 9 Outlooks

With the development of interfacial solar steam/vapor in heating and cooling applications, profound progress has been made in this direction. This review systematically summarizes the background, state‐of‐art progress, challenges, and opportunities of interfacial solar steam/vapor in heating and cooling. Here, we would like to share a few outlooks. Note: The specific outlook for each application has been discussed in the corresponding section. Here, more common issues with opportunities and difficulties will be discussed to avoid repeating the description.

## 10 Heating

For an interfacial solar steam generation used as heating, the biggest challenge is how to achieve high steam temperature while maintaining high conversion efficiency under low‐power sunlight. This requires the development of materials with better performance and better structural design. In the past few years, a series of materials with low thermal conductivity, high absorption, high porosity, hydrophilicity, etc., have been developed to increase the evaporation rate of solar vapor. However, most of these materials are difficult to meet highly efficient heating requirements. For example, at low temperature, the heat radiation loss of the material is not necessary to consider, but in high‐temperature applications, the material with higher absorption, smaller emissivity, and higher high‐temperature stability is needed. So far, selective absorbers with an absorption rate >95% are rarely reported, especially for permeable absorbers. Moreover, like silica aerogel, the materials with high transparency and low thermal conductivity used to improve the performance of photothermal materials are also worthy of further improvement.

From the perspective of devices, apart from rational optical and thermal design to further improve the performances, the intermittent solar light also significantly influences the practical application of heating but has not been looked at thoroughly enough. It is expected that some strategies can be developed to solve this dilemma. Also, we should promote some devices into real applications to explore the different boundaries of the device. The research community should also communicate and collaborate with the industry and the public to explore the possibility of large‐scale production and application. Moreover, exploring some new potential heating applications of interfacial solar steam/vapor generation is also meaningful.

## 11 Cooling

For cooling, the opportunities and challenges of this technology have been discussed in detail in the cooling section. The following three challenges and opportunities for subambient cooling are worthy of our attention. 1) Like other cooling technologies, cooling power is also an important figure of merit in addition to the temperature drop. Therefore, cooling power should be seriously considered in future research. It is worth noting that accurately measuring the cooling power of the absorber, especially for irregular absorbers, is still a challenge. 2) As we know, the process of evaporation is not only related to the properties of the material itself but also strongly related to the external environment. Thus, exploring the relationship between cooling power, temperature drop, material structure, and external weather conditions is also essential. 3) On the application level, developing interfacial solar vapor‐based cooling and subambient cooling's potential application in building energy saving, portable refrigerators, and other fields are also desired.

In summary, it is believed that with the development of new materials and understanding of new mechanisms by researchers, the interfacial solar steam/vapor generation technology can bring new opportunities for effective heating and cooling to alleviate environmental and energy problems.

## Conflict of Interest

The authors declare no conflict of interest.
